# Serotonin: A Potent Immune Cell Modulator in Autoimmune Diseases

**DOI:** 10.3389/fimmu.2020.00186

**Published:** 2020-02-11

**Authors:** Minjie Wan, Lili Ding, Dong Wang, Jiawen Han, Pujun Gao

**Affiliations:** ^1^Department of Hepatology, The First Hospital of Jilin University, Jilin University, Changchun, China; ^2^Central Laboratory, The First Hospital of Jilin University, Jilin University, Changchun, China; ^3^Intensive Care Unit, The First Hospital of Jilin University, Jilin University, Changchun, China

**Keywords:** 5-HT, 5-HT receptor, autoimmune disease, immune cells, serotonin

## Abstract

Serotonin, also known as 5-hydroxytryptamine (5-HT) is a signaling mediator that regulates emotion, behavior, and cognition. Previous studies have focused more on the roles of 5-HT in the central nervous system (CNS). However, 5-HT also shares a strong relationship with the pathological cases of tumor, inflammation, and pathogen infection. 5-HT participates in tumor cell migration, metastatic dissemination, and angiogenesis. In addition, 5-HT affects immune regulation via different 5-HT receptors (5-HTRs) expressed immune cells, including both innate and adaptive immune system. Recently, drugs targeting at 5-HT signaling were tested to be beneficial in mouse models and clinical trials of multiple sclerosis (MS) and inflammatory bowel disease (IBD). Thus, it is reasonable to assume that 5-HT participates in the pathogenesis of autoimmune diseases. However, the underlying mechanism by 5-HT modulates the development of autoimmune diseases has not been fully understood. Based on our previous studies and pertinent literature, we provide circumstantial evidence for an essential role of 5-HT, especially the regulation of 5-HT on immune cells in the pathogenesis of autoimmune diseases, which may provide a new point cut for the treatment of autoimmune diseases.

## Introduction

**Figure 1:**
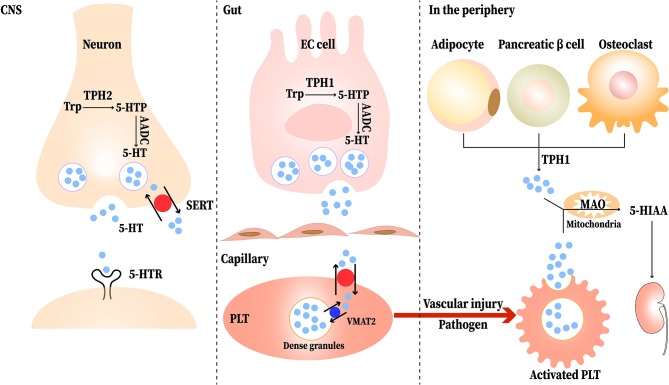
The synthesis and transport of serotonin (5-HT). Neurons and EC (enterochromaffin) cells release 5-HT in response to stimuli. Tryptophan (Trp) is first converted to 5-hydroxytryptophan (5-HTP) via tryptophan hydroxylase (TPH). Then, aromatic amino acid decarboxylase (AADC) immediately converts 5-HTP to 5-HT. The released 5-HT can be transported into neurons and platelets (PLT) via the serotonin reuptake transporter (SERT); most of it is stored in the dense granules of PLT and transported to the peripheral blood. When the PLT are stimulated upon vascular injury or pathogen, the PLT are activated and release the 5-HT to participate physiological reaction. In peripheral tissues, adipocytes, pancreatic β cells, and osteoclasts synthesis 5-HT through TPH1. Completed its physiological function, 5-HT is degraded to 5-hydroxyindoleacetic acid (5-HIAA) by monoamine oxidase (MAO) and then excreted in the urine eventually.

Serotonin, or 5-hydroxytryptamine (5-HT) was first discovered as a vasoconstrictor. It is mainly distributed in the central nervous system (CNS), gastrointestinal (GI) tract, and platelets. About 95% of the 5-HT in our body is synthesized in enterochromaffin (EC) cells of the GI mucosa, and the remaining 5% is produced by serotonergic neurons in the CNS (1). In peripheral tissues, many cells such as adipocytes, pancreatic β cells, and osteoclasts synthesis 5-HT (2). Besides, T cells and mast cells can also produce 5-HT (3). The synthesis pathway of 5-HT contains two enzymatic steps, with tryptophan (Trp) being the primary precursor of 5-HT. Trp is first transformed to 5-hydroxytryptophan (5-HTP) under the action of tryptophan hydroxylase (TPH), including TPH1 and TPH2; most of the TPH1 is found in EC cells and TPH2 is located in the central and enteric neurons. Then, with the present of aromatic amino acid decarboxylase (AADC), 5-HTP is decarboxylated to produce 5-HT (1). Once released, 5-HT is stored and transported in platelets via serotonin reuptake transporter (SERT) and formed the dense granules through vesicular monoamine transporter 2 (VAMT2) and then released in the peripheral blood upon stimulation (such as vascular injury or pathogens) (4). 5-HT can also be absorbed into enterocytes and the vascular endothelial cells via SERT (5). Monoamine oxidase (MAO) degrades 5-HT to 5-hydroxyindoleacetic acid (5-HIAA) after its physiological function is completed (6). The synthesis and transport of 5-HT is shown in Figure 1.

5-HT works primarily by binding to its receptors; seven families (including 15 distinct subtypes, 5-HT_1_-5-HT_7_) of 5-HT receptors have been discovered according to their different signaling mechanisms. 5-HT receptors are the G-protein coupled receptor superfamily all but 5-HT_3_ receptor, which is the Cys-loop ligand-gated ion channel family (7). 5-HT regulates many physiological processes, such as behavior and cognition, including sleep, mood, energy balance, platelet coagulation, tissue regeneration, gastrointestinal function, and immunity (3). The functions of 5-HT in the CNS have been extensively studied, especially the pharmacological manipulations of 5-HT receptors (8). Serotonergic drugs have already been used for the treatment of several kinds of mental disorders such as depression and obsessive-compulsive disorder (9). In addition to its function in the CNS, 5-HT also plays a role in other systems. Accumulating evidence points to the role of 5-HT in the immune system and almost all immune cells express 5-HT receptors. Table 1 lists the 5-HT receptors on different immune cells. 5-HT interacts with the innate as well as the adaptive immune system. In the course of acute inflammation, 5-HT recruits innate immune cells [immature dendritic cells (DCs), monocytes, mast cells and eosinophils, etc.] to the inflammatory site (14). In dextran sodium sulfate (DSS)-induced colitis mice, 5-HT administration increases the release of pro-inflammatory cytokines from macrophages (24). In the collagen-induced arthritis (CIA) mouse, shortage of 5-HT leads to an imbalance of T helper (Th) 17 and T regulatory cells (Tregs) and aggravation of the disease (25). 5-HT_1A_ receptors are more highly expressed on CD4^+^ T cells from multiple sclerosis (MS) patients, and these cells produce more 5-HT than those in healthy individuals (26, 27). Thus, 5-HT is strongly associated with immune cells in autoimmune diseases. This review intends to provide a current understanding of the roles of 5-HT in different autoimmune diseases.

**Table 1:** The expression of 5-HT receptors on immune cells.

**Type**	**Family and subtype**	**References**
Basophils	unknown	
B Cells	1A 2A 3 7	(10)
DC	1B 1E 2A 2B 3 4 7	(11, 12)
Eosinophils	1A 1B 1E 2A 2B 6	(13)
Mast cell	1A	(14)
Macrophages	1A 1B 1E 2A 2B 2C 3 4 7	(15–17)
Monocytes	1E 2A 3 4 7	(17)
Neutrophils	7	(3)
NK	1A 2A 2B 2C	(18, 19)
T Cells	1A 1B 2A 3 7	(20–22)
Platelets	2A 3	(23)

## Digestive System Diseases

### Inflammatory Bowel Disease (IBD)

IBD is a complex inflammatory disorder involving immune dysregulation for an imbalance between pro-inflammatory and anti-inflammatory signaling. Ulcerative colitis (UC) and Crohn's disease (CD) are the major types of IBD. However, the underlying etiology of IBD remains poorly understood (28). The alterations of 5-HT content in the chronic mucosa in both UC and CD have been controversial. Some studies have reported a decrease in 5-HT content, but other experiments have concluded opposite results (29–31). This difference may be related to the degree of mucosal damage. The EC cell population is decreased in severe UC samples compared with that in the non-severe UC group and healthy controls, with there being no difference between the non-severe group and healthy group. Moreover, 5-HT levels are positively correlated with EC cell counts in colonic mucosa biopsies of patients with UC (32). Thus, the changes in 5-HT levels and EC cell numbers in the mucosa in IBD could not be ascertained and depend on the severity of the mucosal damage. These above studies clearly show changes in 5-HT and EC cell levels in the mucosa during the inflammation in IBD; however, it is unclear whether their alterations play any role in gut inflammation.

**Figure 2:**
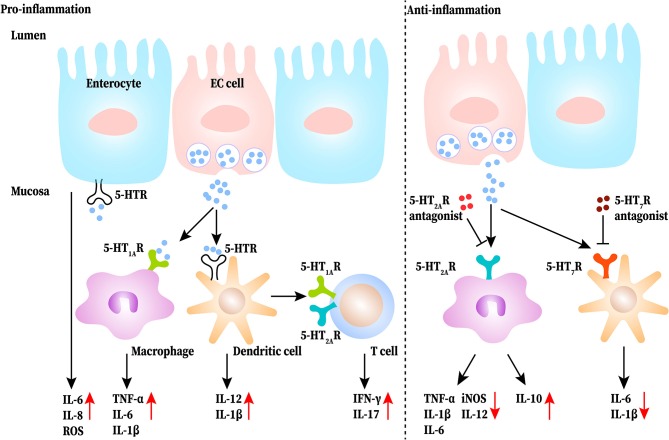
The pro-inflammatory and anti-inflammatory roles of 5-HT signaling pathway in the gut of inflammatory bowel disease (IBD). 5-HT released from EC (enterochromaffin) cells directly or indirectly acts on immune cells (such as macrophages, dendritic cells, and T cells) as well as epithelial cells to produce more proinflammatory cytokines to exasperate gut inflammation. Blocking the 5-HT signaling of macrophages and dendritic cells reduces the release of proinflammatory cytokines and increases IL-10 production.

5-HT may be critical for aggravating symptoms in IBD, including diarrhea and abdominal pain (33). Intraperitoneal administration of 5-HT significantly increases the expression of interleukin (IL)-1β and IL-6 and the activity of myeloperoxidase (MPO) by activating 5-HT_3_ and 5-HT_4_ receptors in colonic mucosa of colitis mice, while blocking the signal can reduce the pain (34). In TPH1 deficient colitis mice, the severity of colitis and the level of IL-1β, IL-6, and tumor necrosis factor (TNF)-α was clearly reduced, and reloading 5-HT increased the severity of DSS-induced colitis (1). A further study reported that 5-HT increased the expression of IL-6, IL-8 and the production of monocyte chemoattractant protein-1 (MCP-1), which lead to the initial events of gut inflammation (35). Thus, 5-HT increased pro-inflammatory cytokine levels in colitis mucosa and exacerbated abdominal pain and colitis in DSS-induced mice. As for the regulation of 5-HT in immune cells, a study by Khan and Ghia demonstrated that 5-HT stimulates macrophages to produce pro-inflammatory cytokines (24). 5-HT also activates dendritic cells (DCs) to produce more IL-12p40 through the NF-κB pathway in the colon of DSS-induced colitis mice, which results in the sequentially increased production of IL-17 and interferon (IFN)-γ by T cells (36). Therefore, 5-HT may play a pro-inflammatory role in the gut which activates DCs, induces T-cell proliferation, sustains immune-cell recruitment, and up-regulates pro-inflammatory cytokine production. Conversely, some studies have demonstrated that 5-HT works as an anti-inflammatory molecule in the GI tract. It has been found that administrating 5-HT_4_ receptor antagonist aggravates disease severity in DSS-induced mice, and the histological damage in the colons of 5-HT_4_ receptor-deficient mice is more severe than that in the control group. The reason may be that the activated 5-HT_4_ receptors can increase epithelial proliferation, promote wound healing, and improve resistance to oxidative stress-induced apoptosis (37). Additionally, 5-HT_2A_ receptors were upregulated in the colons of DSS-induced mice, especially in macrophages. A selective 5-HT_2A_ receptor antagonist, ketanserin, inhibited the expression of CD32 and production of inducible nitric oxide synthase (iNOS) and IL-12 in LPS-macrophages upon LPS challenge, whereas the expression of CD206 and production of IL-10 was increased. This points out that ketanserin may influence macrophages by promoting their anti-inflammatory function and promoting the shift from M1 to M2 through 5-HT_2A_ receptors to exert a protective function (38). Besides, 5-HT receptor antagonists can restrain the production of IL-1β and IL-6 from DCs and alleviate the experimental intestinal inflammation (39). Overall, activation of the 5-HT signaling pathway in the gut has a pro-inflammatory effect and inhibition has an anti-inflammatory effect, but each receptor has a different effect. This functional difference in 5-HT may be due to the diversity of receptors in the GI tract. 5-HT receptors (including 5-HT_1_, 5-HT_2_, 5-HT_3_, 5-HT_4_, and 5-HT_7_ receptors) have been shown to be expressed in the gut (3). Additionally, the function of one type of receptor expressed on different cells such as epithelial or immune cells may be totally different. At the same time, there is a shortage of studies involving the depletion or knockout of a subtype of 5-HT receptor on different immune cells; hence, the role of the 5-HT receptors on different immune cells in a disease state cannot be defined. The pro-inflammatory and anti-inflammatory roles of the 5-HT signaling pathway in IBD are shown in Figure 2.

### Type 1 Diabetes (T1D)

T1D is an autoimmune disease characterized by blood sugar and insulin dysregulation caused by autoimmune damage to the β cells of the pancreatic islets (40). In the non-obese diabetic (NOD) mice, blood 5-HT levels are elevated at 6 weeks after T1D onset and are maintained at high level at all time-points (41). 5-HT can be produced by pancreatic β cells, which complicates the relationship between 5-HT and T1D (42). In T1D, the 5-HT signaling pathway promotes the function and proliferation of β cells. Notably, 5-HT increases the proliferation of the pancreatic β cells in rat insulinoma, and inhibition of 5-HT synthesis blocks β cell expansion (40). Aside from that, the roles of 5-HT in pancreatic islets are diverse. 5-HT enhances β cells to secrete more insulin by activating 5-HT_2_ receptors and 5-HT_3_ receptors. However, the opposite effect of activating 5-HT_1D_ receptors has been reported in healthy human islets and cell lines of β cells, which inhibits insulin secretion (42–44). Thus, the function of 5-HT depends not only on its amount, but also on the receptors it binds. In a hypermetabolic condition such as pregnancy, the increase in β cell mass is due to the prolactin-dependent pathway to induce the expression of TPH1 in β cells, leading to increased production of 5-HT. Then 5-HT activates the intrinsic 5-HT_2B_ receptors and 5-HT_3_ receptors on β cells by autocrine or paracrine signals, leading to β cell proliferation (45, 46). Although 5-HT can interact with β cells directly, the connection between 5-HT and immune cells in T1D is very few. A selective 5-HT_2A_ receptor antagonist, sarpogrelate hydrochloride, reduced macrophages infiltration and down-regulated the expression of NOS2 and TNF-α in diabetes mice (47). Whether 5-HT is associated with the release of pro-inflammatory cytokines released from another immune cells that infiltrate the pancreas in TID needs further study.

Type 2 diabetes (T2D) is a chronic metabolic disease accompanied with insulin resistance and glucoregulatory, usually associated with obesity (48). However, with the further understanding of T2D, considerable evidences point that T2D also had autoimmune components, such as IgG antibodies related to insulin resistance and autoantibodies against pancreatic islets (49, 50). In the early stage of obesity, macrophages and T cells are elevated in adipose tissue. Macrophages and the adaptive immune system are activated even in the absence of pathogenic threat (51). Persistent chronic inflammation in adipose tissue, which in turn promotes inflammation of the systemic system and affects insulin function. Infiltration of macrophages and T cells are found in the islets of T2D (52, 53). To our knowledge, the treatment of SSRIs increases the risk of T2D in depressed patients for SSRIs reducing insulin secretion and increasing insulin dependence (54). A study reported that SSRIs exposure increased ROS and oxidative damage of β cells (55). These strongly suggest that alterations in 5-HT signaling affect T2D disease progression. A Meta-analysis revealed that the risk of T2D in adolescents treated with SSRIs was significantly higher than that in healthy control and psychiatric controls who have not been exposed to SSRIs (56). However, a prospective clinical trial elucidated that T2D patients with depression received sertraline (a type of SSRIs) had lower body weight, body mass index, and waist circumference than those who did not receive treatment (57). Thus, it is optimal to balance the benefits of SSRIs therapy with the increased risk of T2D. And routine indicators such as blood glucose and body weight should be monitored regularly.

### Primary Biliary Cholangitis

PBC is an immune-mediated bile duct destruction and cholestasis, which ultimately leads to liver injury, fibrosis, and cirrhosis. It's the most common chronic cholestatic autoimmune liver disease (58). In animal models of cholestasis with bile duct ligation (BDL), chronic thrombocytopenia aggravates liver fibrosis caused by BDL. Since 5-HT is released by platelets, the same effect can be seen with reduced plasma 5-HT levels (59, 60). In detail, TPH1-deficient mice exhibit higher levels of serum aspartate aminotransferase (AST), alanine aminotransferase (ALT), bile salts, and hepatic necrosis than the WT mice in BDL. But the bile salt reabsorption transporters *Osta* and *Ostb* are up-regulated in the kidneys in TPH1-deficient mice. 5-HT is proposed to down-regulate *Osta* and *Ostb* in the kidneys, causing a decrease in toxic bile salts in the plasma and reducing liver injury (59). Another experiment demonstrated that cholangiocytes express 5-HT receptors as well as TPH2. Cholangiocytes produce 5-HT and inhibit their own growth, but stimulate liver myofibroblasts to produce transforming growth factor (TGF)-β1. Increased TGF-β1 suppresses cholangiocyte growth by inhibiting TPH2 expression in an autocrine manner. In BDL mice with incomplete TPH2 function display reduced biliary 5-HT levels and excessive cholangiocyte proliferation, accumulation of aberrant ductule and liver progenitors, and more severe liver fibrosis, indicating the modulation of cholangiocytes' 5-HT synthesis plays a crucial role in remodeling damaged bile ducts (61). Overall, platelet-derived 5-HT can suppress cholestatic liver injury and biliary fibrosis (59, 62). The number of mast cells which release 5-HT is increased in the skin of PBC (63). A study of BDL rats demonstrated that 5-HT released by mast cells devoted to cholestasis pruritus and 5-HT_3A_ receptor antagonist ondansetron significantly relieved itching symptoms (64). In addition, several clinical trials have observed that ondansetron or SSRIs can be used to alleviate cholestatic itching (65). In the early stage of PBC, NK cell killing activity is enhanced, and monocytes and macrophages have a high response to pathogen-associated antigens (66). Then, Th1 cells and cytotoxic T lymphocyte are activated (67, 68). Furthermore, autoreactive B cells secrete large amount of antimitochondrial antibodies (69). In the final phase, the Th1 reaction shifts to Th17 (70). However, the modulation of these immune cells in the pathological process by 5-HT to exert influence on PBC progression is unknown and needs further research.

## Rheumatic Diseases

### Rheumatoid Arthritis (RA)

RA is a chronic autoimmune inflammatory disease with typical characteristics of chronic inflammation and joint damage (71). Activated platelets and increased serum levels of 5-HT have been reported; high levels may be a negative predictor of bone mineral density in RA for suppressing osteoblasts (72). It has been demonstrated that 5-HT activates 5-HT_1B_ receptors on osteoblasts and inhibits their proliferation by activating proteinkinase A and cAMP-response element binding protein signaling pathway (73). Except for serum, 5-HT increases in RA synovial fluid (74). In the general population, use of SSRIs is related to a higher risk of fractures in patients with RA (75). Therefore, there might be a strong connection between 5-HT and RA. In the CIA mouse model, the content of 5-HT in the paw is increased. Meanwhile, joint inflammation and erosion, bone resorption and osteoclast differentiation, and release of pro-inflammatory factors such as TNF-α, are all promoted in Tph^−/−^ CIA mice (25). In addition to the alteration in local tissues, 5-HT also changes in the hippocampus in the CIA mice because the SERT activity increases and the absorption of 5-HT enhances (76).

Selective 5-HT_3_ receptor antagonists can alleviate arthritic pain and inflammation. In the *in vitro* culture of macrophage-like synovial cells from osteoarthritis (OA), tropisetron can absolutely inhibit 5-HT-induced PGE_2_ release (77). In monocyte *in vitro* cultures from the peripheral blood of healthy donors, the release of TNF-α and IL-1β have been strongly suppressed by tropisetron (78). These data might account for the anti-inflammatory effect of 5-HT_3_ receptor antagonists. Further, the role of T cells in RA has also been reported. Chabbi-Achengli and Comnan indicated that the levels of TNF-α and IFN-γ were increased as well as the level of IL-4 was decreased in TPH1-deficient CIA mice, thus implying a stronger Th1 response. Moreover, the population of Th17 cells increased while that of Tregs decreased in the lymph nodes of CIA mice, and lack of 5-HT caused relative conversion of Tregs to Th17 cells (25). Anti-TNF therapy has been effective in RA, but the treatment of anti-TNF non-responders still remains a challenge. Considering that 5-HT are able to regulate the Th17/Tregs cell balance, the development of therapeutic approaches targeting 5-HT or 5-HT receptors is expected to be a potential prospect for modulating the immune response to RA.

### Systemic Sclerosis (SSc)

SSc is an autoimmune disease characterized by chronic, progressive fibrosis, which affecting the skin and several internal organs, accompanied by abnormal activation of the immune system (79, 80). Plasma 5-HT content is increased in SSc has been reported (81). Another study revealed that intraplatelet 5-HT content is decreased in patients with diffuse SSc compared with that in patients with limited SSc and normal individuals (82). This indicates that the type of SSc also affects the distribution of 5-HT. As previously reported, endothelial cell injury, immune activation, and fibrosis were crucial points in the pathogenetic process of SSc, and 5-HT signaling has been reported to be involved in the pathogenesis of fibrosis, especially the 5-HT/5-HT_2B_ receptor signaling in skin fibrosis (83). Evidently, TGF-β is the most crucial regulatory factor in the pathological fibrosis process of SSc (84). Upon culturing skin fibroblasts with 5-HT from healthy individuals and those with SSc, it was found that 5-HT promoted extracellular matrix proliferation in both conditions through the TGF- β-dependent pathway by activating 5-HT_2B_ receptors, whereas dermal thickening was found to be attenuated with 5-HT_2B_ receptor signaling pathway inhibitors (83). The same conclusion was made in another study. 5-HT administration up-regulated profibrotic genes and collagen production, and treatment with 5-HT_2_ receptor and 5-HT_2B_ receptor antagonists reversed the attenuation of TGF-β1 associated gene expression and collagen production (85).

In addition to the above findings, immune factors, including T cells and B cells, also involve in SSc (86). Effector T cells in SSc are thought to be skewed in a Th2 pattern and key profibrotic mediators (IL-4, IL-6, IL-13) secreted by Th2 cells are a major cause of the fibrosis in SSc (87). However, whether 5-HT affects the course of the disease by affecting immune cells has not yet been reported. Murine basophils were found to participate in the Th2 polarization by instantly secreting lots of IL-4, whereas 5-HT could downregulate this IL-4 production by basophils *in vitro* and *in vivo* (88). Therefore, we hypothesized that 5-HT might inhibit the polarization of T cells to Th2 cells and play an anti-fibrotic role in SSc. This could also explain why Th1 polarization is predominant in most autoimmune diseases, whereas Th2 polarization is predominant in SSc. Further validation by experimental studies is needed.

### Systemic Lupus Erythematosus (SLE)

SLE is an autoimmune rheumatic disorder characterized by multi-system and multi-organ lesions. Inflammation invading sites include the skin, joints, kidneys, and brain (89). The function of 5-HT in the pathogenesis of SLE is currently unclear in spite it's considerable inflammatory effects. The involvement of platelet activation and 5-HT released in SLE pathogenesis has been confirmed (90). A previous study has suggested that decreased platelet size is associated with disease activity in SLE, as activated platelets release several soluble factors, including 5-HT (91). In SLE patients, a study revealed that the 5-HT content decreased in the platelets and increased in plasma, and that these levels are correlated with severity of the disease (92). However, Lood and Tydén demonstrated that the level of 5-HT in both platelets and serum are decreased and no association is found between serum 5-HT levels and clinical disease activity (90). This difference may be related to the patients' disease activity. The recruited SLE patients in the latter study were very few and there were in the inactive period at the time of blood sampling. Also, it may be associated with the activity of indoleamine 2,3-dioxygenase (IDO) (IDO can deprive the ability of Trp for 5-HT synthesis in favor of kynurenine). To support this view, the latter study found that plasmacytoid dendritic cells (pDC) induce large amounts of INF-α, which can increase the expression of IDO and cause the drop of 5-HT in SLE. As for the interaction between 5-HT and immune cells, another study showed that hypomethylation of the 5-HT_1A_ receptor promoter region and high expression of 5-HT_1A_ receptors in the peripheral blood lymphocytes increase the 5-HT level in plasma and lead to the proliferation of T and B cells, which accounts for the process of SLE (21). Similar to other rheumatic diseases, 5-HT is mainly derived from platelets, which plays a role in SLE. But studies on 5-HT in SLE have been limited. This may be related to the disease characteristics of SLE itself. SLE is a disease involving multiple organs, which is not limited to any one site, and is not mainly manifested by the involvement of any one organ.

## Central Nervous System Diseases

### Multiple Sclerosis (MS)

**Figure 3:**
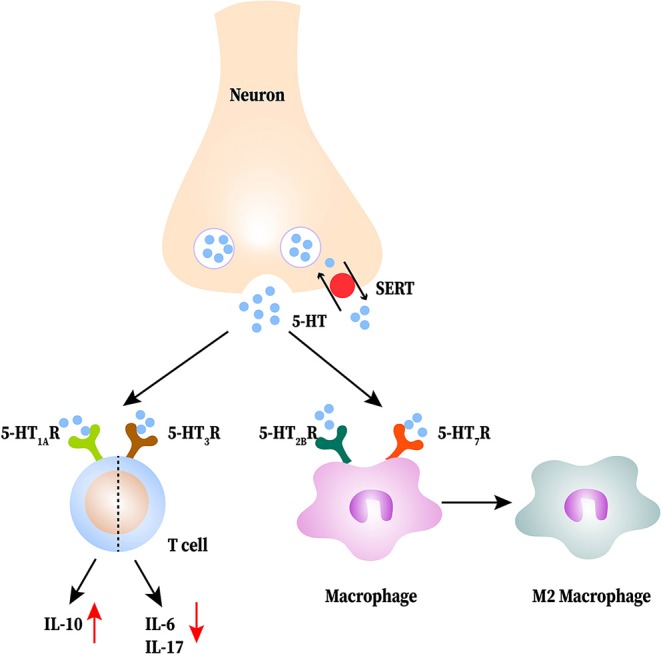
The interactions between serotonin (5-HT) and immune cells in multiple sclerosis (MS). 5-HT released from neurons is reduced in the central nervous system (CNS). 5-HT acts on T cells to produce less proinflammatory cytokines and more IL-10. 5-HT promotes M2 macrophage polarization.

MS is a multiple-site-affected and relapse-remitting alternating disease of the CNS, characterized by myelin and axonal damage (93). Its main features are increased physical disability, cognitive impairment, chronic neuropathic pain, and depression (94). An interesting study showed increased susceptibility of MS in adulthood at high latitudes; it was discovered that the intake of Trp is lower in the high-latitude area than in normal conditions. Lower Trp leads to a decrease of 5-HT resulting in an increased susceptibility of MS; this suggests a potential link between 5-HT and MS (95). In experimental autoimmune encephalomyelitis (EAE) mice, 5-HT content is lower than that in the WT (96). Treating with SSRIs or increasing 5-HT attenuates disease severity, and it is associated with impaired T cell proliferation, lower inflammatory infiltration and IFN-γ production (97). T cells, as wells as macrophages and DCs cells, are generally accepted as the main effectors in MS pathogenesis (26). Higher expression of 5-HT_1A_ receptors in CD4^+^T cells has been detected, which leadings to a 5-HT stimulated increase in IL-10 production by CD4^+^T cells in MS patients (26, 27). *In vitro*, 5-HT suppresses the release of IL-17 and IFN-γ by CD8^+^T cells, both of which are neurotoxic in MS (27). Specifically, IL-17 can induce local (microglia) and migrant (macrophages and DCs) cells to produce more free radicals derived from oxygen and metalloproteinase 9, all of which involved in the neuronal demyelination (98). IFN-γ has been classically considered as the pro-inflammatory cytokine indicating destructive autoimmune T cell activity, including activation of the innate immune system (99). The 5-HT_3_ receptor agonist appears to activate T cells and selective 5-HT_3_ receptor antagonists attenuate disease; this protective effect is related with both reduced *in vitro* IL-6 and IL-17 production by CD4^+^T cells and lower demyelination of the spinal cord (100, 101). In addition to T cells, 5-HT is able to influence macrophage polarization in MS pathological process (93). Through the 5-HT_2B_ receptors and 5-HT_7_ receptors on macrophages, 5-HT makes macrophages prone to polarization to M2 macrophage by inhibition of LPS-induced pro-inflammatory cytokines and regulation of M2 and M1 polarization-related genes (15, 93). Besides, DCs take in and store 5-HT from the local environment and then activate T cells to play a pro-inflammatory role in MS (102). The interactions between 5-HT and immune cells in MS are shown in Figure 3.

Failure to properly control the expression of major histocompatibility complex (MHC) class II on astrocytes may also account to MS (103). In healthy individuals, MHC class II and B7 molecules are not expressed on astrocytes, but these two proteins are detected in the astrocytes at the edges of active MS lesions. Further study has indicated that expressions in astrocytes makes it act as a facultative APC to activate T cells in the CNS (104). Moreover, activation of astrocytic 5-HT_4_ receptors increases the production of intracellular cAMP and inhibits the expression of IFN-γ-mediated induction of MHC class II and B7 co-stimulatory molecules of astrocytes *in vitro* (105). In summary, MS occurs when the content of 5-HT is reduced or its signaling pathway is inhibited in the CNS. A previous study demonstrated that platelets are activated both in the CNS and peripheral blood in MS, and they are recruited into the CNS inflammatory lesions of EAE mice (106). Therefore, we can rationally speculate that it may be the 5-HT reduction in neuronal release or the alteration of SERT expression that affects the MS process rather than the 5-HT released by platelets.

## Others

In addition to the diseases mentioned above, 5-HT has also been studied in other autoimmune diseases. In Hashimoto's thyroiditis mice, IDO1 and SERT are upregulated, accompanied by reduced frontal cortex 5-HT levels. This indicates that alterations in 5-HT signaling in the frontal cortex may be associated with mood control in Hashimoto's thyroiditis patients (107). In Balb/c mice of experimental antiphospholipid syndrome, nervous or mental abnormity such as cognitive deficits and hyperactivity are confirmed to be linked with the up-regulation of 5-HT_1A_ receptors in the hippocampal and cortical regions (108). Additionally, in the imiquimod-induced psoriasis model, mast cells and keratinocytes synthesize 5-HT (109). Further, the percentage of 5-HT-positive cells is significantly higher in skin lesions of psoriasis patients compared to that in normal skin and 5-HT recruits T cells to the inflammatory site of skin and enhance the ability of macrophages as an APC activate T cells (110). Meanwhile, systemic SSRIs treatment has been shown to be beneficial to psoriasis patients from a retrospective cohort study (111). Additionally, a better outcome with SSRIs treatment than placebo from a randomized controlled trial in alopecia areata has been reported (112). These evidences suggest that 5-HT plays a role in various autoimmune diseases, but type of immune cells and mechanisms need further research.

### Interaction Between 5-HT and Th1 and Th2

Th1 cells are important for immunity to intracellular pathogens while Th2 cells are responsible for humoral-mediated immunity. Both of them participate in maintaining immune homeostasis and the variation of them is found in the pathological process of autoimmune diseases (113). 5-HT acted directly on Th1 cells to reduce their production of IFN-γ in MS patients (27). A selective 5-HT_2A_ receptor antagonist, sarpogrelate hydrochloride, inhibited the production of IFN-γ by Th1 cells in a dose-dependent manner *in vitro* culture of mouse spleen CD4^+^T cells, and this inhibition can be reversed by 5-HT_2A_ receptor agonist (22). In addition to the direct effect, activation of 5-HT_2B_ receptor inhibits the polarization of human moDC-primed CD4^+^ T cells toward inflammatory Th1 effector lymphocytes in inflammatory settings (114). How does 5-HT regulate Th2 is poorly studied in autoimmune diseases. But in allergic airway inflammation, lack of 5-HT leads to impaired Th2 priming capacity of DCs (115). However, the alterations of Th1 and Th2 in autoimmune diseases have not been uniformly determined, which may be due to the different subsets playing a leading role in different autoimmune diseases. CD, RA, MS, T1D and Hashimoto's thyroiditis are characterized by dominant Th1 responses while UC and SLE are a Th2-dominated response.

### Interaction Between 5-HT and Th17 and Tregs

The imbalance between Th17 and Tregs has been reported in the literature on autoimmune disease (116–118). Tregs maintain self-tolerance, thereby inhibiting autoimmunity, while Th17 cells promote the induction and propagation of inflammation (116, 117). The balance between these two types of CD4^+^T cells—one promoting inflammation, the other controlling the adaptive immune responses play an important role in autoimmune diseases (118). There is a strong association between 5-HT and these two types of cells in autoimmune diseases. In EAE mice, Th17 cells activate microglia to produce IL-6, IL-1β, and TNF-α. Then, IL-6 inhibits the synthesis of 5-HT by reducing tetrahydrobiopterin and TNF-α activates IDO, which breaks down Trp to reduce 5-HT level in the brain (119). In the *in vitro* culture of peripheral blood mononuclear cells isolated from MS patients, 5-HT reduces the production of IL-17 and IFN-γ from Th17 (27).

**Figure 4:**
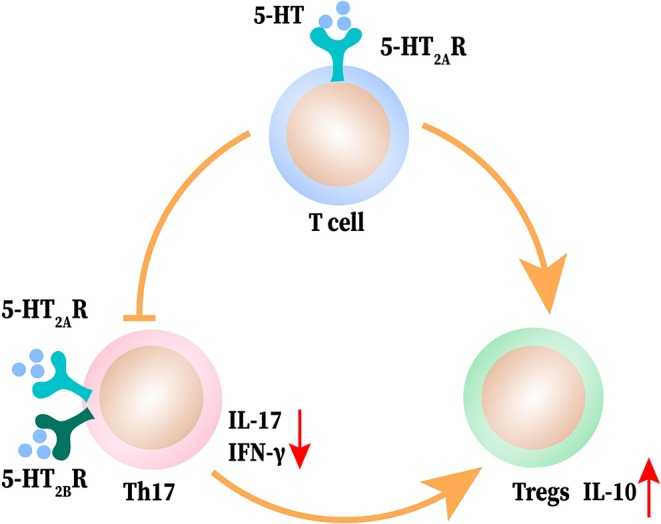
The interaction between 5-HT and T helper (Th) 17 and T regulatory cells (Tregs). 5-HT induces T cell differentiation to Treg cells and promotes the shift of Th17 cells to Tregs. 5-HT acts on Th17 to produce less IFN-γ and IL-17 and elevates the release of IL-10 from Tregs.

5-HT-deficient mice displayed a relatively, dampened expansion of Tregs accompanied with an increased shift toward a Th17 phenotype in arthritis (120). Similarly, in Tph1^−/−^ mice of CIA, the proportion of Tregs is reduced (Foxp3^+^CD25^high^CD4^+^T cells, to be precise) and Th17 is increased. Reloading 5-HT can reverse the polarization of T cells to Th17 and reduce the production of IL-17 through 5-HT_2A_ and 5-HT_2B_ receptors on the surface of T cells. Meanwhile, 5-HT activates the 5-HT_2A_ receptors on CD4^+^ T non-Tregs to promote Tregs proliferation (25). 5-HT upregulates regulatory marker CD39 on the surface of CD4^+^T cells, increases IL-10 production, and enhances Tregs to inhibit T effector cells proliferation (27). After ischemic stroke, 5-HT can promote the proliferation of Tregs in the brain along with the expression of Tregs surface markers and reduce neurological dysfunction; this effect can be blocked by 5-HT_7_ receptor antagonists (121). In addition to Th17 and Tregs, Treg 17 is a novel regulatory T cell subset that co-expresses IL-17 and IL-10. 5-HT up-regulates the proportion of non-classical regulatory Th17 cells (27). Overall, 5-HT can inhibit Th17 differentiation and IL-17 production, promote Tregs phenotypic expression, inhibitory capacity and proliferation, and regulate the balance of Th17/Tregs to maintain immune homeostasis in autoimmune disease and inflammatory conditions. The interactions between 5-HT, Th17, and Tregs are shown in Figure 4.

### Interaction Between 5-HT and Macrophage Polarization

Macrophage polarization plasticity is critical for maintaining tissue homeostasis. 5-HT has been reported to involved in macrophage polarization mechanisms (15). *In vitro* culture of human monocytes, SSRIs inhibited the release of LPS-induced pro-inflammatory cytokine. The same effect was observed with activation of 5-HT_4_ and 5-HT_7_ receptor on monocytes (122). Additionally, another study demonstrated that during the generation of monocyte-derived macrophages, 5-HT had this inhibitory capacity without affecting the output of IL-10, and up-regulated the M2 polarization-related gene through activating 5-HT_2B_ and 5-HT_7_ receptors, which tended to express on M2 macrophage (15). In DSS-induced colitis mice, 5-HT_2A_ receptor was higher expressed on intestinal macrophages. With the treatment of a selective 5-HT_2A_ receptor antagonist, ketanserin, intestinal inflammation was alleviated, along with M2 polarization, lower pro-inflammatory cytokine production, and impaired migration of macrophage. Knockout of the 5-HT_2A_ receptor abrogated this anti-inflammatory effect and inhibited the NF- κB pathway in macrophages. Therefore, 5-HT may regulate macrophage polarization through 5-HTR2A/NF-κB (38). Considering the capacity of 5-HT to promote M2 polarization of macrophages, inhibition of 5-HT may become a new breakthrough point in the treatment of neuroendocrine tumors (NETs). A clinical case report described telotristat (TPH inhibitor) can play an antitumor role to significantly relief the carcinoid syndrome symptoms and improve the quality of life of NETs (123). Collectively, 5-HT regulates the polarization of macrophages through multiple receptors and pathways, including both activated and inhibitory signals. In the inflammatory condition or tumor microenvironment, 5-HT promoting M2-polarization of macrophages may become a new treatment direction.

## Conclusions

In spite of its recent discovery, there is accumulating evidence that 5-HT plays significant roles in autoimmune diseases. The effect of 5-HT on immune cells depends on the cell type, 5-HT receptor subtype, as well as the disease itself. Due to these factors, it is difficult to define the pro- or anti-inflammatory roles of 5-HT. In this review, we build a framework linking 5-HT alterations to autoimmune diseases through its effects on the immune cells. It has been confirmed that 5-HT regulates the balance of Th17/Tregs and promotes M2-polarization of macrophages and has a direct or indirect regulatory effect on traditional immunocytes such as T cells, macrophages, DC cells, and NK cells. However, little research about the interaction of 5-HT and some novel immune cells has been reported. Recently, regulatory B cells have attracted widespread attention for their function in autoimmune diseases such as IBD and MS (124, 125). Besides, innate lymphoid cells have been considered as regulators of immunity, inflammation, and tissue homeostasis (126). Therefore, there are several unanswered questions. For example, is 5-HT associated with these new immune cells? Are there any undiscovered 5-HT receptor subtypes? Is the expression and distribution of 5-HT receptors on immune cells comprehensive? In the same autoimmune disease, can 5-HT acting on different immune cells have different or even opposite roles? In autoimmune diseases, can 5-HT regulate immune cells through other receptors or pathways, besides 5-HT receptors? What are the differences in the mechanisms by which 5-HT functions in the CNS and in other systems? Elucidating these mysteries may provide a better understanding of the roles of 5-HT in autoimmune diseases.

The role of 5-HT as a neurotransmitter in the brain has been widely studied. A range of 5-HT modulating drugs such as SSRIs, monoamine oxidase inhibitors (MAOI), tricyclic antidepressants (TCA), and 5-HT norepinephrine reuptake inhibitors (SNRI) are designed to treat neurological diseases. Additionally, these drugs can also be applied in some autoimmune diseases. In a large and robust cohort, depression increased the risk of developing both CD and UC. The use of SSRIs or other antidepressants protected against CD and UC (127). It has been demonstrated that patients with IBD are more likely to suffer depression, and that depression worsens the prognosis of IBD (128). In addition to IBD, the clinical trial of fluoxetine has been carried out in secondary-progressive MS (129). Therefore, various evidences have shown that 5-HT and autoimmune diseases are inextricably linked. Targeting the 5-HT signaling pathway may be a new potential prospect in the treatment of autoimmune diseases.

## Author Contributions

MW wrote the manuscript. LD and DW consulted relevant literature. JH drew the figures. PG revised the manuscript.

### Conflict of Interest

The authors declare that the research was conducted in the absence of any commercial or financial relationships that could be construed as a potential conflict of interest.
